# Supplementary material for: Collaborative intelligence in AI: Evaluating the performance of a council of AIs on the USMLE
Source: PLOS Digit Health. 2025 Oct 9;4(10):e0000787. doi: 10.1371/journal.pdig.0000787 (PMC12510544; doi:10.1371/journal.pdig.0000787)
Supplement: S2 File — (ZIP) [file pdig.0000787.s002.zip › S2 Data Raw data files containing unprocessed question inputs and LLM outputs including transcripts of deliberations/STEP3_Question-47-1_Type-MC-NJ.docx]

**Facilitator** **[Round 1]:** A 60-year-old man comes to the office because of a 1-year history of progressive left groin pain. The pain is worse with exercise and is relieved with rest. He says the pain is beginning to affect his ability to carry out his daily activities. He says, ""It even hurts when I'm turning over in bed."" He does not recall any trauma to the area. He also has had generalized mild stiffness of his joints during the past 3 years. He takes atorvastatin for hyperlipidemia and lisinopril for hypertension. Medical history is otherwise unremarkable. He does not smoke cigarettes. He drinks one alcoholic cocktail daily. He is 178 cm (5 ft 10 in) tall and weighs 68 kg (150 lb); BMI is 22 kg/m². Vital signs are temperature 37.4°C (99.3°F), pulse 82/min, respirations 18/min, and blood pressure 120/78 mm Hg. The patient walks with a slight limp and has difficulty changing position. Abduction of the left hip elicits pain. Physical examination of the patient's other joints discloses no abnormalities. Which of the following is the most appropriate imaging study at this time? (A) CT scan of the hips (B) MRI of the hips (C) Radionuclide bone scan (D) Plain x-ray of the left hip (E) Skeletal survey

**AI_B:** RESTATING THE QUESTION: The narrative describes a 60-year-old man experiencing pain in his left groin over a year. This pain is exaggerating with physical activity and easing with rest. He's also experiencing pain whilst turning over in bed, which negatively affects his daily life. He has a mild stiffness in joints over the last three years. On walking, he limps and the manoeuvre of left hip-abduction evokes pain. His daily medications include atorvastatin for his high cholesterol and lisinopril for high blood pressure. His medical history doesn't reveal anything notable. This man neither smokes nor has any issues with abnormal vitals. The challenge here is choosing the most appropriate imaging method to diagnose his condition among CT scan, MRI, Radionuclide bone scan, plain X-ray, or a skeletal survey. PLANNING: The main assumption here should be what condition might cause a man to develop progressive left groin pain exacerbated by movement and improved at rest. To explore further, factors that could result in such symptoms include degenerative joint disease (such as osteoarthritis), hip bursitis, hip labral tear, or hip osteonecrosis. Exercise-induced pain that improves with rest, difficulty in changing positions, stiffness of joints, and pain with a passive range of motions suggest more of mechanical nature, usually seen in degenerative arthritis, or osteoarthritis. It is a chronic process of cartilage destruction, joint space narrowing, subchondral sclerosis, and osteophyte development. For this condition, Plain X-ray is usually the first and best modality to visualize bony abnormalities. CLINICAL REASONING: Osteoarthritis, being the likely medical condition, typically presents with joint pain that worsens with use and improves with rest, which matches our patient's symptoms. Although other hip-related conditions could cause these symptoms, the overall clinical picture, including his age, paint a comprehensive picture of osteoarthritis. Based on this assumption, the most beneficial imaging study to evaluate osteoarthritis would be a plain x-ray of the left hip. It can reveal characteristic osteoarthritic changes such as joint space narrowing, subchondral sclerosis, and osteophytes. CONCLUDING: Taking all these into consideration, the most appropriate imaging study for this patient would be Plain X-ray which allows for cost-effective and quick evaluation of his hip joint. Later, as needed, further studies can be pursued based on X-ray findings. RESPONSE: Therefore, the most appropriate imaging study to carry out for this patient at this time is the Plain X-ray of the left hip (option D).

**AI_E:** (1) RESTATING THE QUESTION: In more simple terms, the question presents the case of a 60-year-old man who's been experiencing left groin pain for a year, which worsens with activity and is improving with rest. He is also reporting difficulty in performing his day to day activities due to this pain. There are no history of any trauma to the area and he is having a slight problem with his movements due to the pain. Also, he experiences some difficulty while trying to change his position. His overall medical history and lifestyle habits suggest no significant clues towards the diagnosis. Further, the physical examination has shown pain upon abduction (overhead or sideways movement) of his left hip. With these facts taken into consideration, the question is asking for the most appropriate imaging examination at this point. (2) PLANNING: The patient has localized pain causing disability without any trauma history, suggesting a degenerative cause rather than a traumatic one. An infectious cause could be ruled out due to absence of fever, redness, swelling, or systemic signs of infection. Neoplastic causes can be considered but are less likely due to the lack of systemic symptoms such as weight loss or other specific symptoms. So we are assuming that the most probable cause here might be a degenerative or mechanical problem, likely related to osteoarthritis. Therefore, it will be beneficial to have an imaging study that allows us to evaluate the anatomical structures well, especially the joint surfaces in this case. (3) CLINICAL REASONING: With the hypothesis being a degenerative or mechanical problem, we should first go for cheaper, less invasive, and sufficiently effective imaging options. Plain x-ray is a good starting point which provides a good view of bones, can identify degenerative changes such as osteoarthritis, deformities, large masses, and some other bone abnormalities. (4) CONCLUDING: Highlighting the facts that the patient shows hip pain, especially with activation, and has a reduced ability, it is likely that the pain originates from a problem with his hip joint. Given the non-traumatic, non-infectious, and non-neoplastic nature of his condition, a degenerative problem becomes a high possibility. Out of all available imaging options, an X-ray will be the initial choice to evaluate for any signs of osteoarthritis and other bone problems. (5) RESPONSE: Therefore, the most appropriate imaging study at this time is a 'Plain x-ray of the left hip' (option D).

**AI_A:** (1) RESTATING THE QUESTION: A 60-year-old man presents with a history of increasing left groin pain over the past year, which intensifies with physical activity and offers some relief upon resting. No trauma is recalled, but he has experienced broad mild joint stiffness over three years. Taking medications for high blood lipids (hyperlipidemia: explained by atorvastatin) and high blood pressure (hypertension: explained by lisinopril), he leads a moderate lifestyle with no other notable medical history. His vital statistics and physical profile are within normal limits. However, he walks with a mild limp due to pain upon movement and hip abduction, without other joint abnormalities on medical examination. The task is to identify the optimal imaging examination for this patient's condition. (2) PLANNING: Let's take into consideration the patient's symptoms, pain worsened by exercise and relieved by rest, indicating a potential functional issue or degenerative condition. Considering his age, this could suggest the possibility of a musculoskeletal disease such as osteoarthritis. We have to decide between a CT scan (A), MRI (B), radionuclide bone scan (C), a plain x-ray of the left hip (D) or a skeletal survey (E). Each method offers different advantages in imaging bone and soft tissue structures. (3) CLINICAL REASONING: A CT scan (A) provides good resolution for bony structures but may not be as helpful in evaluating for soft tissue conditions. An MRI (B), while excellent for evaluating soft tissue structures, might not be necessary at this initial evaluation stage, given the specific location and the nature of the pain which suggests the hip joint as the primary site of pathology. The radionuclide bone scan (C) is an option when looking for subtle, occult bone lesions, especially when there are multiple suspicious areas, which seems less likely given the patient's specific left groin symptoms. A skeletal survey (E), a series of x-rays of all the bones in the body, is usually not indicated for localized pain. (4) CONCLUDING: That leaves us with the plain x-ray of the left hip (D). It's a highly appropriate first diagnostic image for this patient presenting with isolated left hip pain that is worse with physical activity and improves with rest, could quite likely be due to a degenerative bone condition such as osteoarthritis. Furthermore, an x-ray is an easily accessible, minimally invasive, and cost-effective way to evaluate for signs of joint degeneration such as bony spurs or reduced joint space, which are common in osteoarthritis. (5) RESPONSE: Hence, the most appropriate imaging study at this time for this patient would be a plain X-ray of the left hip (D). It will be helpful to evaluate the possible presence of osteoarthritis or other degenerative joint conditions. If there are abnormalities seen on the x-ray or if the x-ray does not match the clinical picture, other imaging modalities such as MRI could be considered at a later stage.

**AI_C:** (1) RESTATING THE QUESTION: The question is concerning a 60-year-old man presenting with a year-long history of worsening left groin pain that is aggravated by physical activity and relieved by rest. He also experiences discomfort when moving in bed. Concurrently, he has been experiencing generalized mild joint stiffness for the past three years. Despite his lifestyle and medical regimen for hyperlipidemia and hypertension, physical examination reveals pain upon the abduction of the left hip but no other joint abnormalities. In light of his symptoms and examination findings, one must decide the most appropriate imaging technique between a CT scan of his hips, an MRI of the hips, a radionuclide bone scan, a plain x-ray of the left hip, and a full skeletal survey. (2) PLANNING: Various assumptions can be made based on the man's clinical history and physical examination, such areas of focus could be that; there may be arthritis, potential hip osteoarthritis, or even some form of hidden trauma. With each assumption, the most clinically relevant imaging study could differ. If arthritis is suspected, an imaging study such as an MRI might be more suitable to visualise soft tissues such as cartilage and synovial inflammation often associated with arthritis. If hip osteoarthritis is contemplated due to his age and the nature of his symptoms, the most appropriate imaging would be a plain x-ray of the left hip, as this can show changes related to osteoarthritis like narrow joint space, subchondral sclerosis, and osteophyte formation. If concealed trauma is suspected, CT scans might be more suggestive due to its higher resolution of bony structures. (3) CLINICAL REASONING: Connecting these considerations logically, given his age of 60 years and the described symptoms of hip pain that worsens with activity and relieves with rest, coupled with the pain during abduction of the hip, his condition points towards osteoarthritis of the hip joint, which is a common condition in older adults. Furthermore, his additional symptom of generalised joint stiffness might be an early indicator of age-related degenerative changes across other joints too, stressing the likelihood of osteoarthritis. (4) CONCLUDING: Given these deductions, the most appropriate imaging study to confirm this assumption and provide useful information about the state of the left hip joint would be a plain x-ray. This type of imaging allows for the visualization of joint space narrowing, subchondral sclerosis, and the formation of osteophytes, which are characteristic findings in osteoarthritis. CT scans, MRIs, and radionuclide bone scans, while more sophisticated, may not provide this necessary information and can sometimes even complicate the diagnostic process. (5) RESPONSE: Therefore, in this clinical scenario of the 60-year-old man presenting with features suggestive of hip osteoarthritis, the most clinically appropriate imaging study to undertake would be (D) a Plain x-ray of the left hip.

**AI_D:** 1) RESTATING THE QUESTION: The medical case here concerns a 60-year-old man who has been experiencing progressive left groin pain for a year, which worsens with exercise and eases with rest. This pain is beginning to disrupt his daily activities, especially as now it hurts even when he's turning over in bed. He also has a 3-year history of generalized mild stiffness of his joints. He has no known history of trauma in that area. His medications include atorvastatin for high cholesterols and lisinopril for high blood pressure. Other aspects of his medical history are quite normal, and he's not a smoker but takes one alcoholic cocktail daily. His physical examination reveals difficulty in walking and changing position along with elicited pain during left hip abduction. Based on these symptoms and physical findings, we're tasked to identify the most appropriate imaging study at this time, choosing from options such as CT scan, MRI, radionuclide bone scan, plain x-ray, and skeletal survey. 2) PLANNING: This requires making assumptions. The symptoms like groin pain that worsens with activity and is relieved with rest, combined with difficulty in changing positions and the patient's age could suggest hip osteoarthritis. Another assumption could be hip bursitis given his age and symptomatic presentation. If it's a hip osteoarthritis case, an x-ray would be the most useful initial imaging study since it could reveal reduced joint space, osteophytes, and subchondral sclerosis. If it's hip bursitis, ultrasound would probably be the most beneficial test (not among the options here). These assumptions can be made based on standard diagnostic guidelines and the patient's clinical presentation. 3) CLINICAL REASONING: An important symptom pointing towards an osteoarthritis diagnosis is patient's progressive pain over one year, which worsens with exercise. This goes along with the physical examination finding of pain on abduction, which is a hallmark of hip involvement in osteoarthritis. Other conditions such as hip bursitis or a stress fracture would not have bothered the patient for such a long duration, and the patient is not reporting symptoms of inflammation like fever or unbearable pain. Another factor supporting the osteoarthritis diagnosis is his age. Osteoarthritis commonly affects the weight-bearing joints in individuals aged 50 or more. 4) CONCLUDING: Given that osteoarthritis is a degenerative joint disease primarily affecting weight-bearing joints like the hips, it's most likely that the patient's one-year history of left groin pain, notably worse with exercise and relieved with rest, is due to hip osteoarthritis. Our assumptions and clinical reasoning turns the focus towards osteoarthritis. Thus, the first step in imaging study, in line with current guidelines, should be a plain x-ray. 5) RESPONSE: Therefore the most appropriate imaging study at this time for this 60-year-old patient with a one-year history of left groin pain, is a (D) Plain x-ray of the left hip. This will confirm whether the suspected hip osteoarthritis is present through characteristic features such as reduced joint space or osteophytes.

**Facilitator** The consensus among the AI responses is that the most appropriate imaging study for this 60-year-old man, who presents with a year-long history of progressive left groin pain that intensifies with physical activity and eases with rest, is a plain x-ray of the left hip (option D). This conclusion is based on the patient's symptoms, age, and physical examination findings, which suggest a degenerative or mechanical issue, likely osteoarthritis. A plain x-ray is a cost-effective, minimally invasive, and efficient initial imaging study that can reveal characteristic osteoarthritic changes such as joint space narrowing, subchondral sclerosis, and osteophytes. While other imaging modalities like CT scans, MRIs, and radionuclide bone scans can provide more detailed views of the bone and soft tissue structures, they may not be necessary at this initial evaluation stage, given the specific location and nature of the patient's pain. If the x-ray results are inconclusive or do not align with the clinical picture, more advanced imaging studies could be considered at a later stage.
